# Supplementary material for: Doublet microtubule-associated tektins and enzymes differentially regulate sperm flagellar integrity and motility
Source: Nat Commun. 2026 Feb 28;17:3316. doi: 10.1038/s41467-026-69714-4 (PMC13066092; doi:10.1038/s41467-026-69714-4)
Supplement: Supplementary file 1 — Supplementary_Information [file 41467_2026_69714_MOESM1_ESM.docx]

**Supplementary information**

**Doublet Microtubule-Associated Tektins and Enzymes Differentially Regulate Sperm Flagellar Integrity and Motility**

Qi Liu^1,8^, Lunni Zhou^2,3,4,5,8^, Xiaochen Liang^2,3,4,5,8^, Pengyu Chen^1^, Bo Li^1^, Shuo Yang^1^, Yuqi Liu^1^, Haibo Zhao^1^, Jin Hu^3,6^, Shan Feng^3,6^, Shanshan Xie^7^, Jianping Wu^2,3,4,5*^ Miao Gui^1*^

^1^Department of Obstetrics and Gynecology, Sir Run Run Shaw Hospital and Liangzhu Laboratory, Zhejiang University School of Medicine, Hangzhou 310058, Zhejiang, China

^2^State Key Laboratory of Gene Expression, School of Life Sciences, Westlake University, Hangzhou 310024, Zhejiang, China

^3^Zhejiang Key Laboratory of Structural Biology, School of Life Sciences, Westlake University, Hangzhou 310024, Zhejiang, China

^4^Westlake Laboratory of Life Sciences and Biomedicine, Hangzhou 310024, Zhejiang, China

^5^Institute of Biology, Westlake Institute for Advanced Study, Hangzhou 310024, Zhejiang, China

^6^Mass Spectrometry & Metabolomics Core Facility, The Biomedical Research Core Facility, Westlake University, Hangzhou 310024, Zhejiang, China.

^7^Children's Hospital, Zhejiang University School of Medicine, National Clinical Research Center for Child Health, Hangzhou 310052, Zhejiang, China

^8^These authors contributed equally

^*^These authors jointly supervised this work

Correspondence: wujianping@westlake.edu.cn (J.W.), miaogui@zju.edu.cn (M.G.)

**Contents**

Supplementary Figures 1-7

Supplementary Tables 1-4

**Supplementary Figures**

**
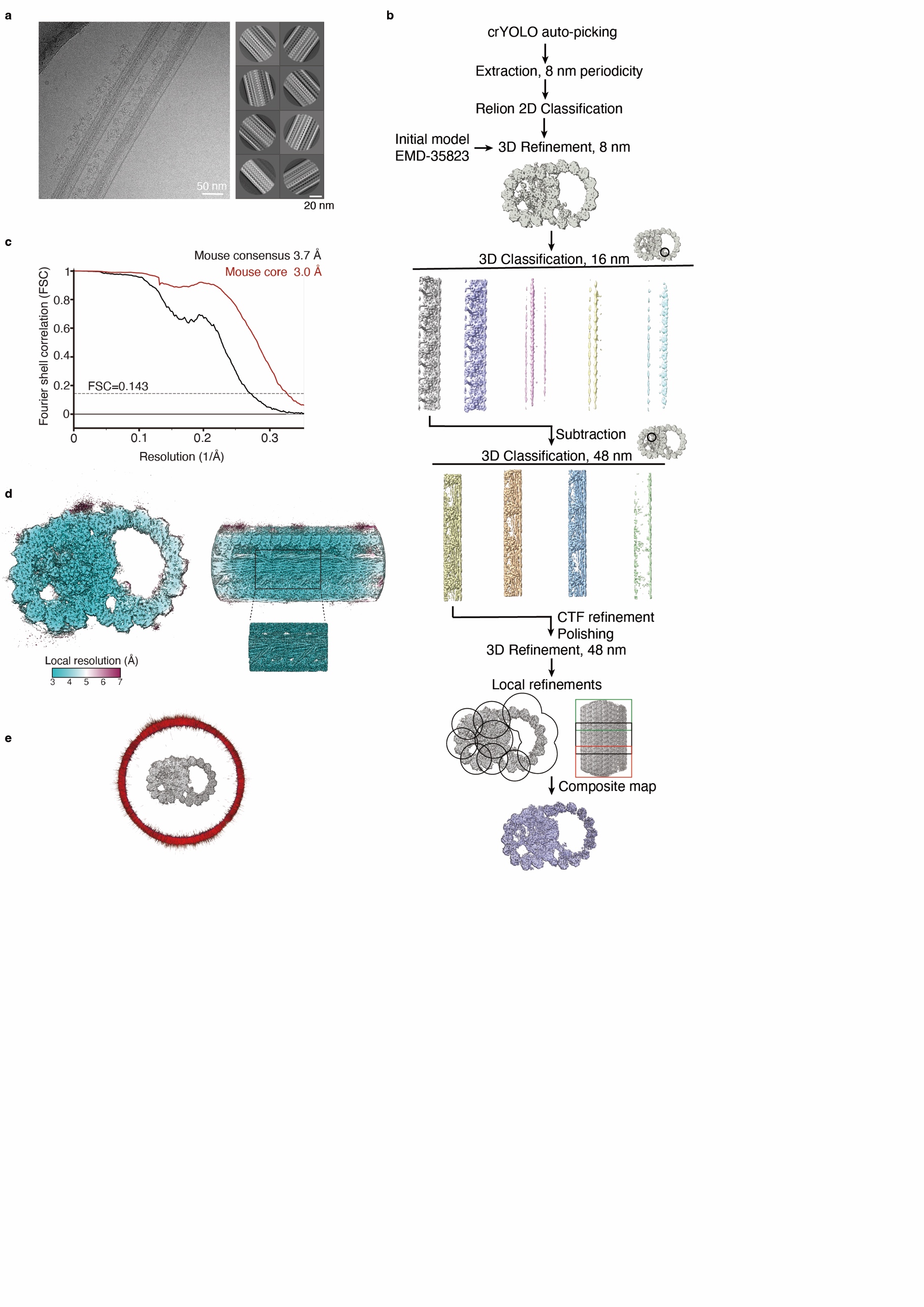
**

**Supplementary Figure 1. Cryo-EM data processing of WT mouse sperm DMTs.**

(a) Cryo-EM micrographs (left) and 2D class averages (right) of mouse sperm DMTs.

(b) Flow diagram showing the processing strategy of 48-nm repeat density map of mouse sperm DMT.

(c) FSC curves for mouse sperm DMT consensus map and local refined map of the core region.

(d) Consensus map of mouse sperm DMT colored by local resolution. The inserted figure shows the local refined maps of the corresponding regions colored by local resolution.

(e) Particle orientation distributions around the density maps.

**
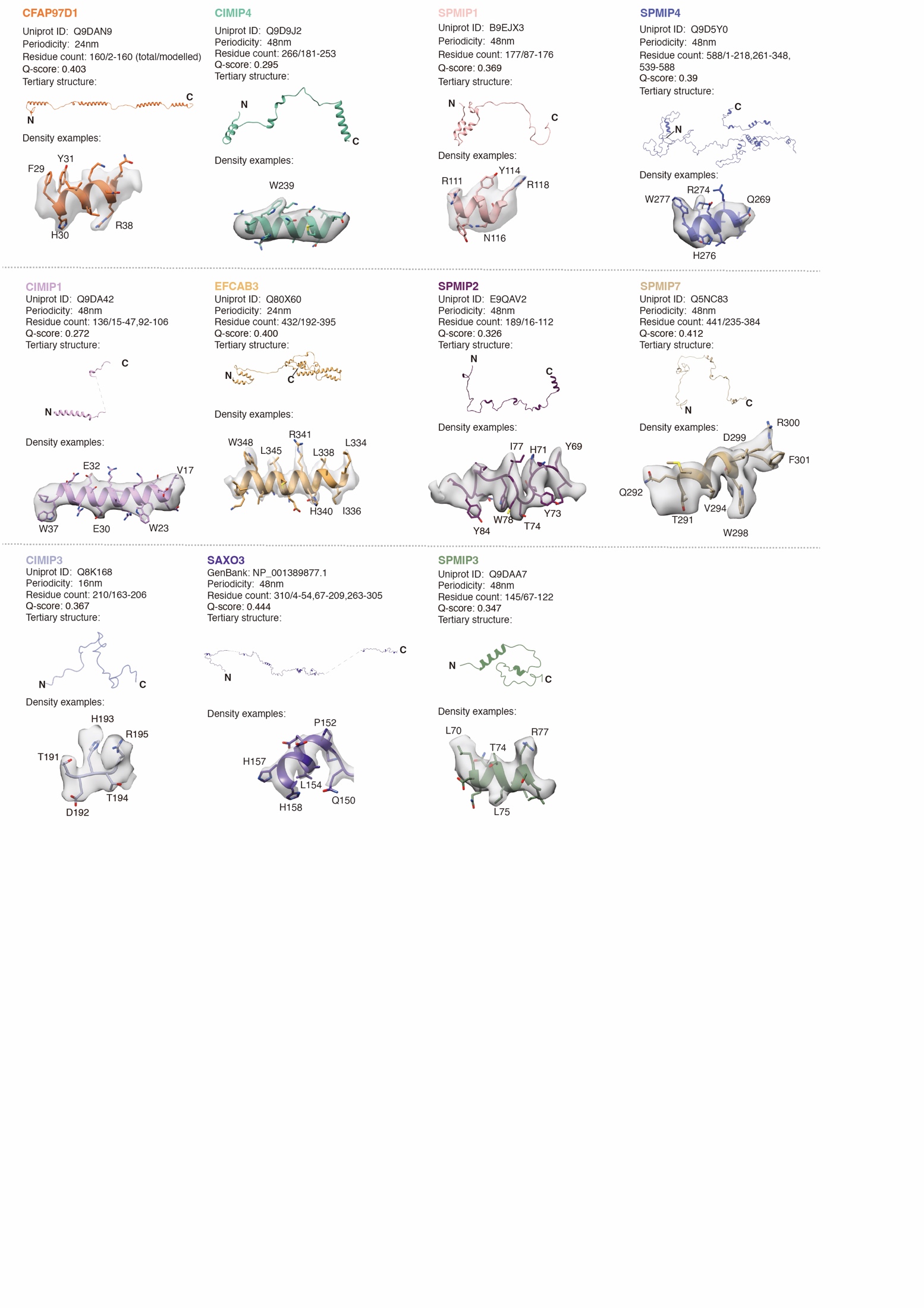
**

**Supplementary Figure 2. Newly identified mouse sperm DMT-associated proteins.**

Density maps around the atomic models of selected regions are shown. Landmark residues are presented.

The source data underlying Supplementary Fig. 2 are provided as a Source Data file.

**
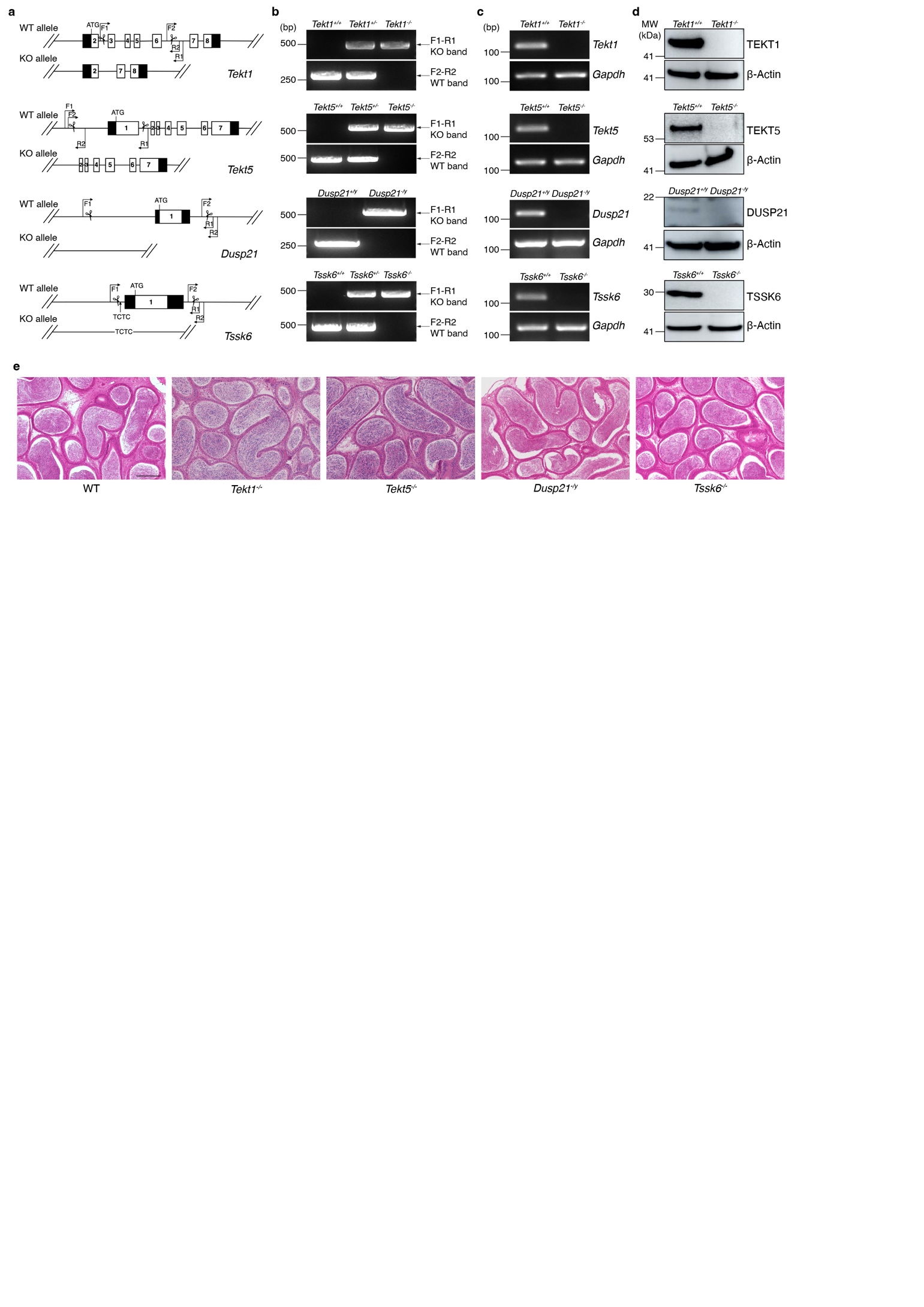
**

**Supplementary Figure 3. Generation and validation of knockout mice.**

(a) Strategies for generating knockout mice targeting *Tekt1*, *Tekt5*, *Dusp21* and *Tssk6*, respectively. The WT allele and the corresponding KO allele after editing were shown. Filled boxes: noncoding regions, boxes with numbers: exons, scissors: the CRISPR/Cas9 gene editing sites. The start codon ATG and the locations of primers (F1, R1, F2 and R2) used for genotyping were labeled. F1 and R1 are outside the targeting region. F2 and R2: one is inside the targeting region and the other is outside the targeting region.

(b) PCR genotyping of KO male mice using genomic DNA as templates. Images are amplified PCR products after electrophoresis with F1 and R1 indicating the knock-out bands (upper) and with F2 and R2 indicating the WT bands (lower).

(c) RT-PCR validation of mRNA expression in testes from the indicated mice using the directly inverse-transcripted cDNA as templates. Images are amplified PCR products after electrophoresis with the gene-specific primers (upper) and with primers of *Gapdh* as a control (lower).

(d) Western blot validation of the protein expression in cauda epididymal spermatozoa from the indicated mice. β-actin is used as a control.

(e) HE staining of cauda epididymal sections from WT and KO mice. Scale bar: 200 µm.

The source data underlying Supplementary Fig. 3 are provided as a Source Data file.

**
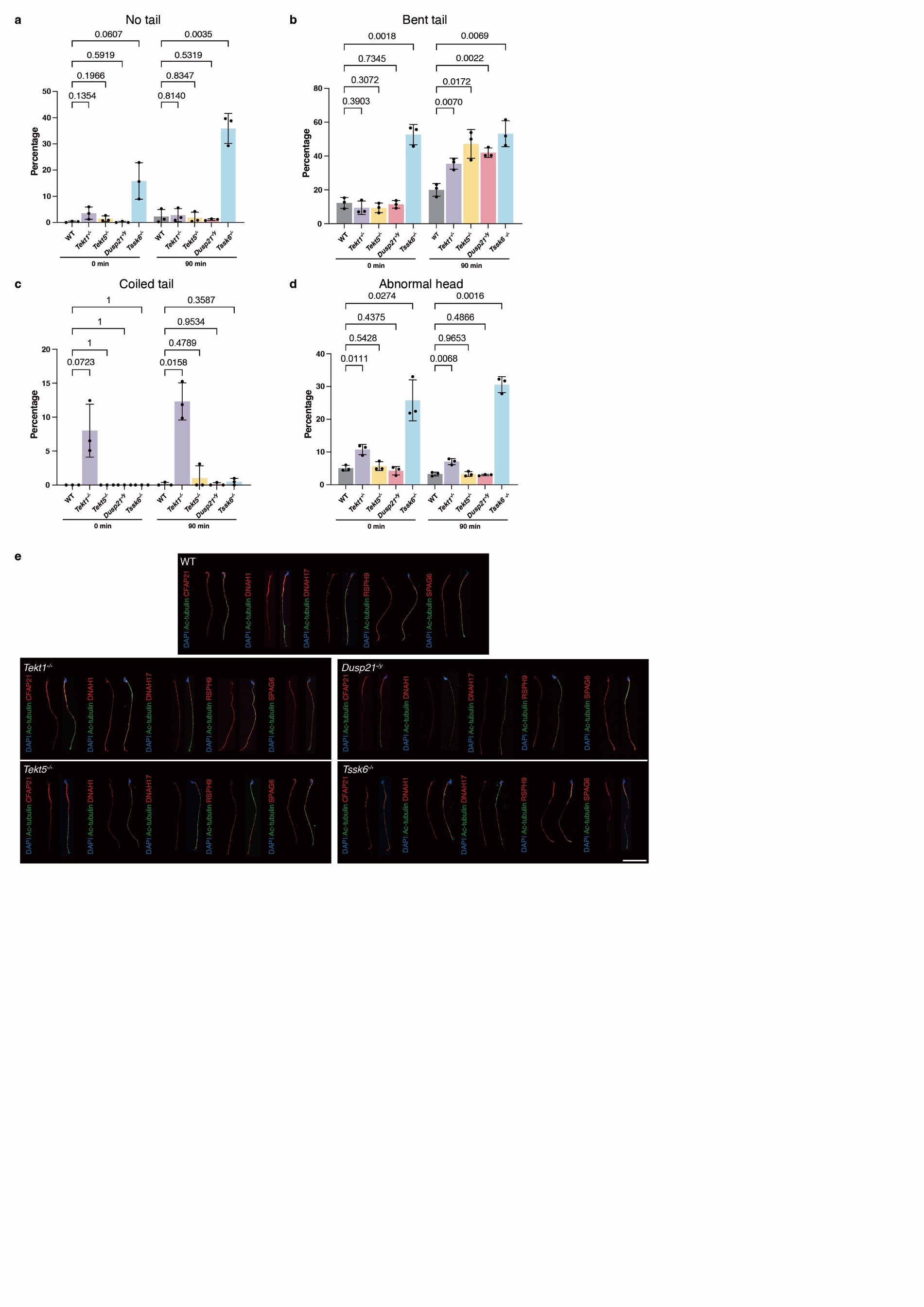
**

**Supplementary Figure 4. Morphological statistics and immunofluorescence staining of spermatozoa.**

(a-d) Statistics of tail (a-c) and head (d) morphology of cauda epididymal spermatozoa from indicated male mice before (0 min, left) and after (90 min, right) capacitation, including no tail (a), bent tail (b) and coiled tail (c). n=3 males. Over 200 spermatozoa from each male mouse are counted. Data are presented as mean ± SD. Statistical significance was assessed using an unpaired two-tailed *t*-test.

(e) Immunofluorescence staining of spermatozoa from WT, *Tekt1*^-/-^, *Tekt5*^-/-^, *Dusp21*^-/y^, and *Tssk6*^-/-^ mice using anti-Ac-tubulin (green), anti-CFAP21 (MIP, red), anti-DNAH1 (IDA, red), anti-DNAH17 (ODA, red), anti-RSPH9 (RS, red), anti-SPAG6 (N-DRC, red) antibodies. The nucleus is stained with DAPI (blue). Scale bar: 40 μm.

The source data underlying Supplementary Fig. 4 are provided as a Source Data file.

**
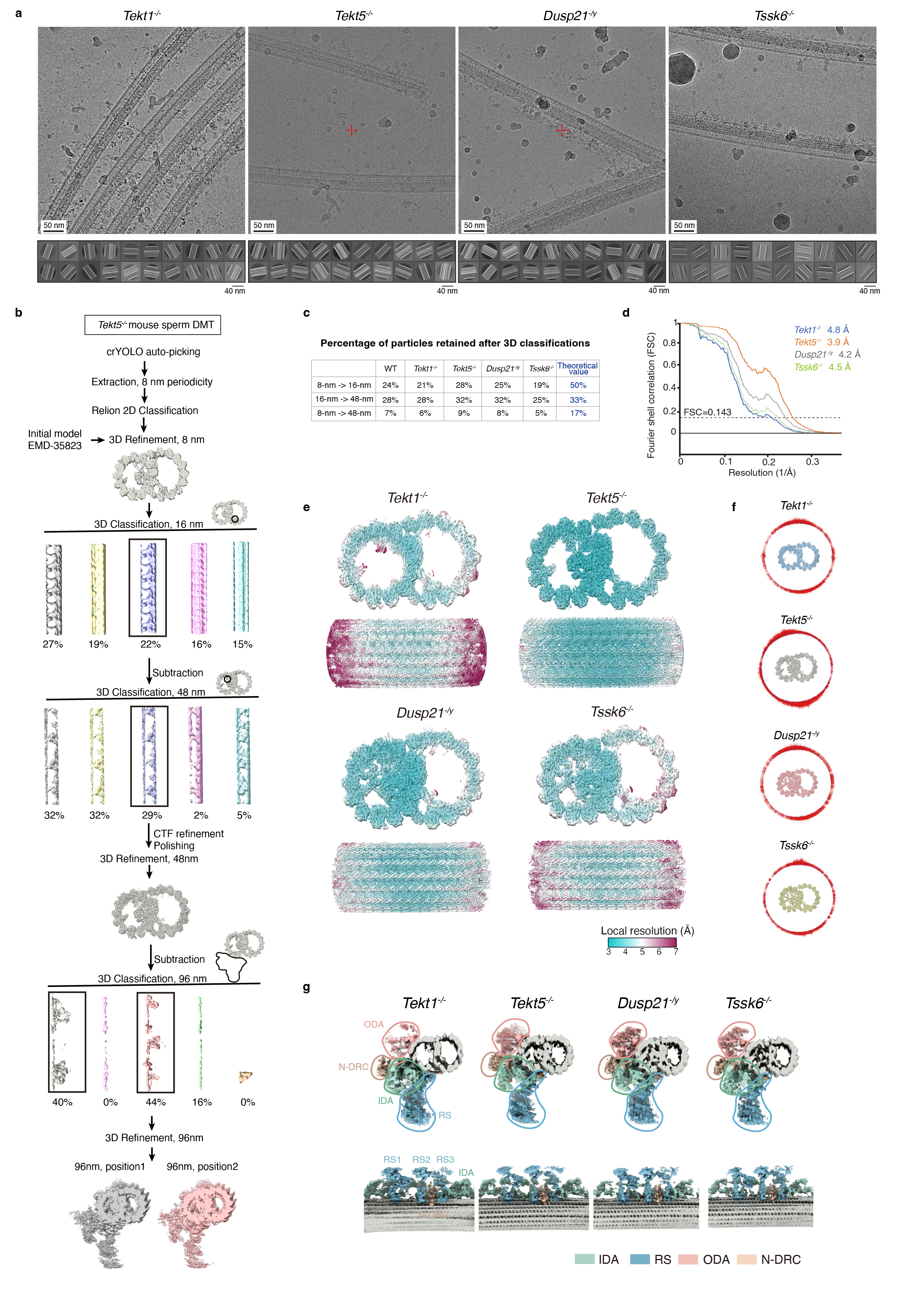
**

**Supplementary Figure 5. Cryo-EM data processing of knockout mouse sperm DMTs.**

(a) Representative cryo-EM micrographs and 2D class averages of *Tekt1^-/-^, Tssk6*^-/-^ and *Dusp21^-/y^*. Scale bar: 50nm.

(b) Flow diagram showing the processing strategy of 48-nm repeat density map of *Tekt5^-/-^* DMTs. Data processing of *Tekt1^-/-^, Tssk6*^-/-^ and *Dusp21^-/y^* DMTs are similar.

(c) Percentage of particles retained after 3D classification compared with the theoretical value.

(d) FSC curves for the KO mouse sperm DMT consensus maps.

(e) Consensus maps of KO mouse sperm DMTs colored by local resolution.

(f) Particle orientation distributions around the density maps.

(g) 96-nm density maps of the KO mouse sperm DMTs showing the presence of external axonemal complexes including ODA, IDA, RS and N-DRC.

The source data underlying Supplementary Fig. 5 are provided as a Source Data file.


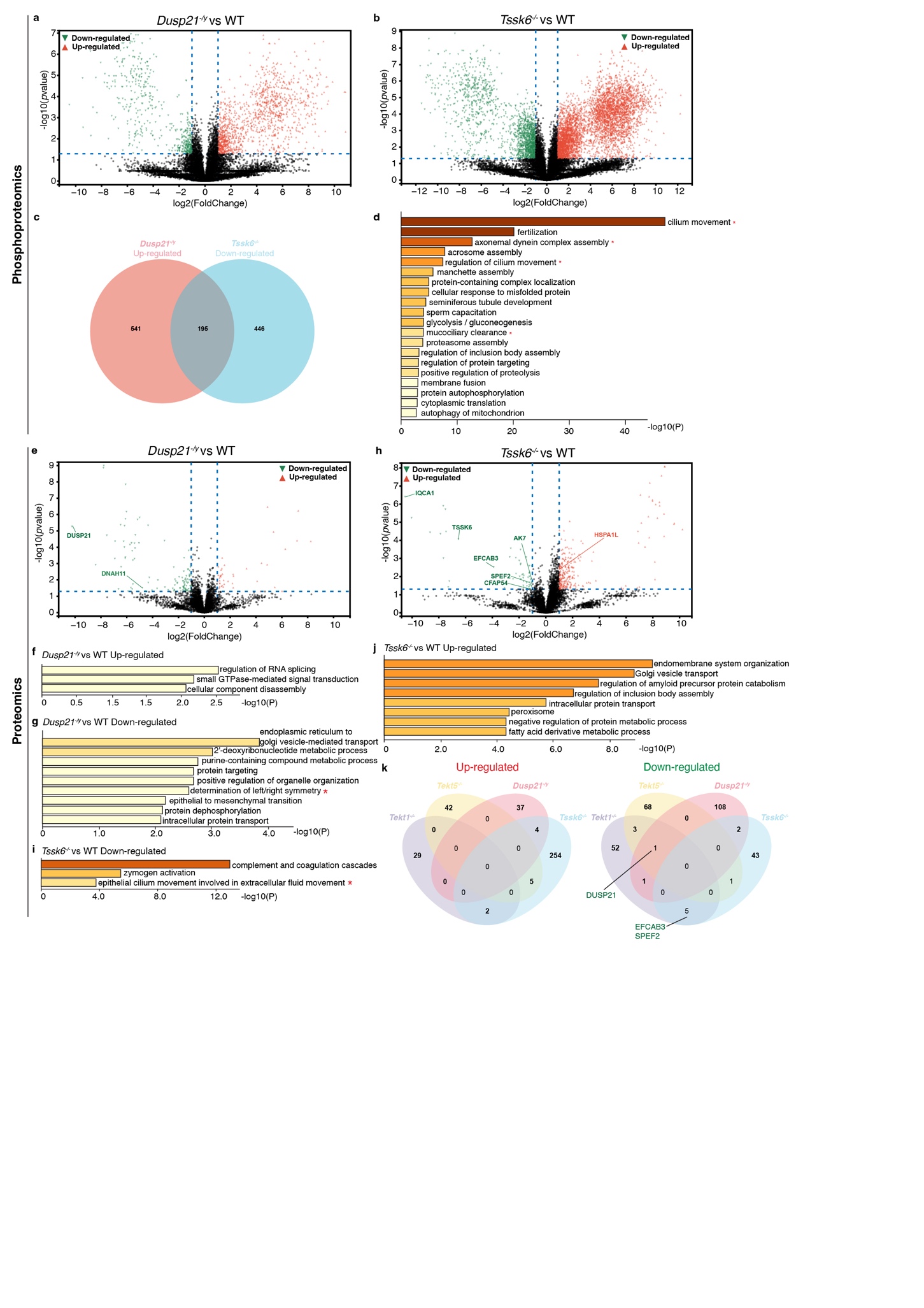


**Supplementary Figure 6. Quantitative proteomics and phosphoproteomics.**

(a,b) Volcano plot shows the differentially regulated phosphorylation sites of *Dusp21*^-/y^ (A) and *Tssk6*^-/-^ spermatozoa (b) compared with WT (best localization probability ≥ 0.95, fold change ≥ 2, FDR < 0.05). Red triangles represent up-regulated phosphorylation sites and green triangles represent down-regulated phosphorylation sites. Statistical significance was assessed using an unpaired two-tailed *t*-test, and *p*-values were adjusted for multiple testing using the Benjamini-Hochberg procedure to control the FDR.

(c) Venn diagram shows the overlap of up-regulated phosphorylated proteins of *Dusp21^-/y^* spermatozoa and down-regulated phosphorylated proteins of *Tssk6*^-/-^ spermatozoa. The numbers in each section represent the count of differentially regulated proteins that are unique or shared among different KO spermatozoa.

(d) GO and KEGG analyses of the shared proteins in (c). Terms related to flagellar motility and assembly are marked with red asterisks. Statistical significance for term enrichment was assessed using a hypergeometric test, and *p*-values were adjusted for multiple testing using the Benjamini-Hochberg procedure to control the FDR.

(e) Volcano plot shows the differentially expressed proteins in *Dusp21^-/y^* spermatozoa compared with WT (fold change ≥ 2, *p*-value < 0.05). Statistical significance was assessed using an unpaired two-tailed *t*-test.

(f,g) GO and KEGG analyses of up-regulated (f) and down-regulated (g) proteins in *Dusp21*^-/y^ spermatozoa compared with WT. Terms related to flagellar motility and assembly are marked with red asterisks. Statistical significance for term enrichment was assessed using a hypergeometric test, and *p*-values were adjusted for multiple testing using the Benjamini-Hochberg procedure to control the FDR.

(h) Volcano plot shows the differentially expressed proteins in *Tssk6*^-/-^ spermatozoa compared with WT. Statistical significance was assessed using an unpaired two-tailed *t*-test.

(i,j) GO and KEGG analyses of up-regulated (i) and down-regulated (j) proteins in *Tssk6*^-/-^ spermatozoa compared with WT. Terms related to flagellar motility and assembly are marked with red asterisks. Statistical significance for term enrichment was assessed using a hypergeometric test, and *p*-values were adjusted for multiple testing using the Benjamini-Hochberg procedure to control the FDR.

(k) Venn diagrams illustrate the overlaps of up-regulated or down-regulated proteins among *Tekt1*^-/-^, *Tekt5*^-/-^, *Dusp21*^-/y^ and *Tssk6*^-/-^ spermatozoa. The numbers in each section represent the count of differentially expressed proteins that are unique or shared among different KO spermatozoa. Proteins related to flagellar assembly or motility are labeled.

The source data underlying Supplementary Fig. 6 are provided as a Source Data file.

**
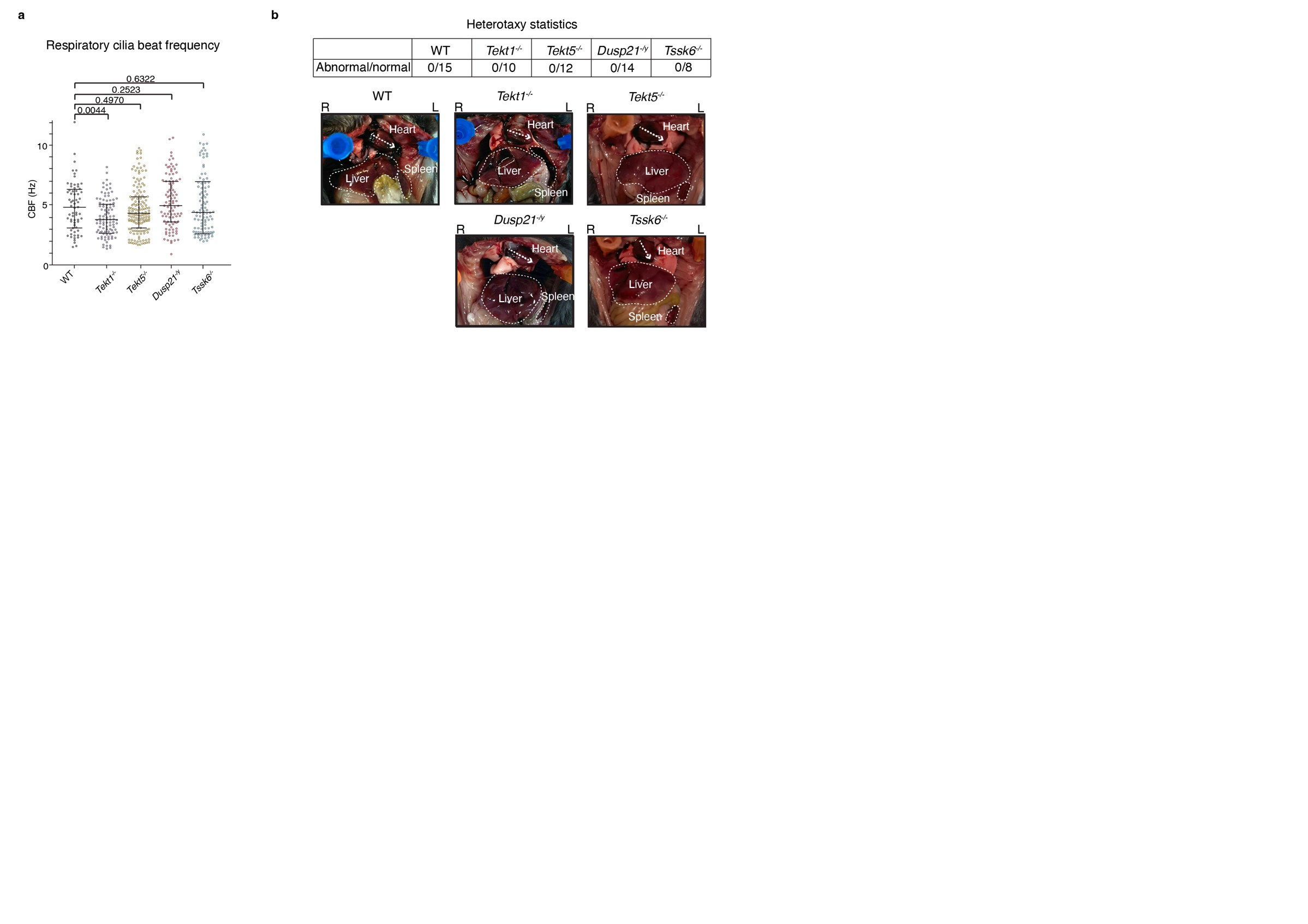
**

**Supplementary Figure 7. Function of respiratory cilia and nodal cilia.**

(a) Beat frequency of mouse respiratory cilia from WT and KO male mice. Statistical significance was assessed using an unpaired two-tailed *t*-test.

(b) Heterotaxy analyses of WT and KO male mice. Representative images of organ positions from WT and KO mice were shown. Heart, liver and spleen were labeled. All the observed animals show normal laterality.

The source data underlying Supplementary Fig. 7 are provided as a Source Data file.

**Supplementary Table 1. Cryo-EM data collection, refinement, and validation statistics.**

|  | 48-nm repeat of wild-type mouse sperm DMT  (EMD-64679)  (PDB 9V10) | 48-nm repeat of *Tekt1*^-/-^ mouse sperm DMT  (EMD-64623) | 48-nm repeat of *Tekt5*^-/-^ mouse sperm DMT  (EMD-64624) | 48-nm repeat of *Dusp21*^-/y^ mouse sperm DMT  (EMD-64626) | 48-nm repeat of *Tssk6*^-/-^mouse sperm DMT  (EMD-64625) |
| --- | --- | --- | --- | --- | --- |
| **Data collection** |  |  |  |  |  |
| Microscope | Titan Krios | Titan Krios | Titan Krios | Titan Krios | Titan Krios |
| Detector | K3 | Falcon 4i | K3 | Falcon 4i | Falcon 4i |
| Voltage (keV) | 300 | 300 | 300 | 300 | 300 |
| Nominal magnification | 81,000 x | 130,000 x | 81,000 x | 130,000 x | 130,000 x |
| Electron exposure (e^-^/Å^2^) | 50 | 50 | 50 | 50 | 50 |
| Defocus range set during data acquisition (µm) | -1.3 to -2.8 | -1.5 to -2.5 | -1.5 to -2.6 | -1.5 to -2.5 | -1.5 to -2.5 |
| Pixel size (Å) | 1.087 | 0.93 | 1.087 | 0.93 | 0.93 |
| **Data Processing** |  |  |  |  |  |
| Initial 8-nm particles | 1,614,369 | 842,367 | 1,217,339 | 1,467,446 | 611,877 |
| Final 48-nm particles | 83,043 | 26,075 | 83,599 | 22,430 | 25,926 |
| Map resolution (Å) | 3.7 | 4.8 | 3.9 | 4.2 | 4.5 |
| **Model composition** |  |  |  |  |  |
| Chains | 589 |  |  |  |  |
| Atoms | 1,603,403 |  |  |  |  |
| Residues | 201,106 |  |  |  |  |
| Ligands | 340 |  |  |  |  |
| **Refinement** |  |  |  |  |  |
| Resolution limit set in refinement (Å) | 4 |  |  |  |  |
| Correlation coefficient (CCmask) | 0.66 |  |  |  |  |
| Root-mean-square deviation (bond lengths) (Å) | 0.012 |  |  |  |  |
| Root-mean-square deviation (bond angles) (Å) | 1.161 |  |  |  |  |
| **Validation** |  |  |  |  |  |
| MolProbity Score | 1.94 |  |  |  |  |
| Clashscore | 12.79 |  |  |  |  |
| Rotamer outliers (%) | 0.65 |  |  |  |  |
| Ramachandran (favored) (%) | 95.42 |  |  |  |  |
| Ramachandran (allowed) (%) | 4.51 |  |  |  |  |
| Ramachandran (outliers) (%) | 0.07 |  |  |  |  |

**Supplementary Table 2. Raw data of computer-assisted sperm analysis (CASA).**

|  |  | **5 min** | | **90 min** | |
| --- | --- | --- | --- | --- | --- |
| **Strain** | **Mouse** | **Motile cells (%)** | **Progressive cells (%)** | **Motile cells (%)** | **Progressive cells (%)** |
| **WT** | 1 | 75 | 40 | 69 | 39 |
|  | 2 | 82 | 44 | 66 | 33 |
|  | 3 | 86 | 45 | 66 | 43 |
|  | 4 | 74 | 38 | 64 | 30 |
|  | 5 | 78 | 37 | 78 | 36 |
|  | 6 | 67 | 28 | 73 | 36 |
|  | 7 | 58 | 25 | 66 | 31 |
|  | 8 | 77 | 35 | 75 | 42 |
|  | 9 | 79 | 36 | 67 | 36 |
|  | 10 | 78 | 33 | 73 | 43 |
| ***Tekt1^-/-^*** | 1 | 25 | 4 | 19 | 5 |
|  | 2 | 29 | 7 | 25 | 7 |
|  | 3 | 30 | 6 | 34 | 11 |
|  | 4 | 25 | 4 | 39 | 12 |
|  | 5 | 28 | 4 | 38 | 12 |
|  | 6 | 36 | 8 | 45 | 12 |
| ***Tekt5^-/-^*** | 1 | 52 | 20 | 63 | 30 |
|  | 2 | 65 | 27 | 74 | 38 |
|  | 3 | 73 | 29 | 67 | 31 |
|  | 4 | 72 | 30 | 74 | 41 |
|  | 5 | 71 | 25 | 73 | 39 |
| ***Dusp21^-/y^*** | 1 | 79 | 41 | 65 | 27 |
|  | 2 | 74 | 33 | 68 | 23 |
|  | 3 | 79 | 36 | 66 | 27 |
|  | 4 | 74 | 36 | 61 | 28 |
|  | 5 | 91 | 43 | 71 | 33 |
|  | 6 | 77 | 33 | 68 | 31 |
|  | 7 | 77 | 32 | 85 | 51 |
| ***Tssk6^-/-^*** | 1 | 31 | 10 | 9 | 3 |
|  | 2 | 20 | 7 | 8 | 2 |
|  | 3 | 20 | 7 | 10 | 4 |
|  | 4 | 29 | 10 | 8 | 4 |
|  | 5 | 29 | 12 | 11 | 5 |
|  | 6 | 31 | 12 | 10 | 4 |

**Supplementary Table 3. Absent proteins in sperm DMT of KO mice by cryo-EM analyses.** Red-labeled proteins indicate the absence of depleted genes. Partially lost proteins in the cryo-EM maps are marked in gray.

| **48-nm repeat of *Tekt1*^-/-^ mouse sperm DMT** | **48-nm repeat of *Tekt5*^-/-^ mouse sperm DMT** | **48-nm repeat of *Dusp21*^-/y^ mouse sperm DMT** | **48-nm repeat of *Tssk6*^-/-^mouse sperm DMT** |
| --- | --- | --- | --- |
| DUSP21 | DUSP21 | DUSP21 | CFAP97D1 |
| SAXO3 | TEKT5 | TEX37 101-125 | EFCAB3 |
| SAXO4 | TEX37 101-180 |  | TSSK6 |
| TEKT1 | TEKT3 81-92 |  |  |
| TEKT2 | FAM166A (near tektin) 39-84, 91-104, 175-188  FAM166A (near A05) 210-260 |  |  |
| TEKT3 |  |  |  |
| TEKT4 |  |  |  |
| TEKT5 |  |  |  |
| TEKTIP1 |  |  |  |
| FAM166A (near tektin) 39-104, 175-188  FAM166A (near A05) 210-260 |  |  |  |
| FAM166C 104-135 |  |  |  |
| SPMIP6 191-214 |  |  |  |
| TEX37 101-180 |  |  |  |

**Supplementary Table 4. Primers for genotyping of KO mice.**

| **Mouse strain** | **Primer** | **Sequence** |
| --- | --- | --- |
| *Tekt1*^-/-^ | Forward primer 1 (F1) | 5’-GGGTGTAGATCAGCGACAGAGTATAT-3’ |
|  | Reverse primer 1 (R1) | 5’-CAGTTCGTTATGAATTAGCGTTGG-3’ |
|  | Forward primer 2 (F2) | 5’-TGCATACAGGACCCTGTGTAGTAGC-3’ |
|  | Reverse primer 2 (R2) | 5’-ACGAGAACTGGAGGTTGTACTTCCC-3’ |
| *Tekt5*^-/-^ | Forward primer 1 (F1) | 5’-TTGGAATATGTAACACAGCAGTCCAC-3’ |
|  | Reverse primer 1 (R1) | 5’-TCCACCTCTCGTCTCCATGTGT-3’ |
|  | Forward primer 2 (F2) | 5’-ACTGCTAAAGATGGTAGAGCCTAAGG-3’ |
|  | Reverse primer 2 (R2) | 5’-TCCAGGAAGCTGACACCACACTT-3’ |
| *Dusp21*^-/y^ | Forward primer 1 (F1) | 5’-CTGTGGGAGCTTTTATAGTAAACCTAG-3’ |
|  | Reverse primer 1 (R1) | 5’-GACTCACTTTGCCTGCTCTTATGG-3’ |
|  | Forward primer 2 (F2) | 5’-AGACCTGCCAATAGCATCATGTG-3’ |
|  | Reverse primer 2 (R2) | 5’-AGTCAGTGTCCATGAGAGGAATCC-3’ |
| *Tssk6*^-/-^ | Forward primer 1 (F1) | 5’-ACCTGAGCACCATCACTTCAGG-3’ |
|  | Reverse primer 1 (R1) | 5’-CTTCTCAAAATCCCAATATCTGGTTC-3’ |
|  | Forward primer 2 (F2) | 5’-AACCGGAGTAGGGAGATTCAAATCC-3’ |
|  | Reverse primer 2 (R2) | 5’-CACGGGAGTTCCTCACAACGTAC-3’ |
