# Supplementary material for: Doublet microtubule-associated tektins and enzymes differentially regulate sperm flagellar integrity and motility
Source: Nat Commun. 2026 Feb 28;17:3316. doi: 10.1038/s41467-026-69714-4 (PMC13066092; doi:10.1038/s41467-026-69714-4)
Supplement: Supplementary file 2 — Description of Additional Supplementary Files [file 41467_2026_69714_MOESM2_ESM.pdf]

**Title:** Supplementary Video 1

**Description**: High-speed video microscopy recordings of mouse spermatozoa. (A) An example of normal sperm beating from a wild-type mouse. (B-E) Examples of sperm beating from a *Tekt1*$^{-/-}$ (B), *Tekt5*$^{-/-}$ (C), *Dusp21*$^{-/y}$ (D) or *Tssk6*$^{-/-}$ (E) mouse. Videos were recorded at 200 frames per second using a Nikon Spinning Disk CSU-W1 SoRa microscope equipped with camera ORCA-Fusion BT (HAMAMATSU). Videos are displayed at 25 frames per second. All mice were between 2-4 months of age.

**Title**: Supplementary Video 2

**Description**: High-speed video microscopy recordings of mouse tracheal cilia. **(**A) An example of normal ciliary beating from a wild-type mouse. (B-E) Examples of ciliary beating from a *Tekt1*$^{-/-}$ (B), *Tekt5*$^{-/-}$ (C), *Dusp21*$^{-/y}$ (D) or *Tssk6*$^{-/-}$ (E) mouse. Videos were recorded at 200 frames per second using an Olympus IX83 microscope equipped with a 60× magnification objective and a high-speed camera. Videos are displayed at 25 frames per second.
