# Supplementary material for: Doublet microtubule-associated tektins and enzymes differentially regulate sperm flagellar integrity and motility
Source: Nat Commun. 2026 Feb 28;17:3316. doi: 10.1038/s41467-026-69714-4 (PMC13066092; doi:10.1038/s41467-026-69714-4)
Supplement: Supplementary file 5 — Video 2 [file 41467_2026_69714_MOESM5_ESM.pdf]

nature portfolio

Corresponding author(s): Miao Gui

Last updated by author(s): Jan 21, 2026

Reporting Summary

Nature Portfolio wishes to improve the reproducibility of the work that we publish. This form provides structure for consistency and transparency in reporting. For further information on Nature Portfolio policies, see our Editorial Policies and the Editorial Policy Checklist.

Statistics

For all statistical analyses, confirm that the following items are present in the figure legend, table legend, main text, or Methods section.

n/a	Confirmed	
☐	☒	The exact sample size (n) for each experimental group/condition, given as a discrete number and unit of measurement
☐	☒	A statement on whether measurements were taken from distinct samples or whether the same sample was measured repeatedly
☐	☒	The statistical test(s) used AND whether they are one- or two-sided<br>*Only common tests should be described solely by name; describe more complex techniques in the Methods section.*
☒	☐	A description of all covariates tested
☒	☐	A description of any assumptions or corrections, such as tests of normality and adjustment for multiple comparisons
☐	☒	A full description of the statistical parameters including central tendency (e.g. means) or other basic estimates (e.g. regression coefficient) AND variation (e.g. standard deviation) or associated estimates of uncertainty (e.g. confidence intervals)
☐	☒	For null hypothesis testing, the test statistic (e.g. F, t, r) with confidence intervals, effect sizes, degrees of freedom and P value noted<br>*Give P values as exact values whenever suitable.*
☒	☐	For Bayesian analysis, information on the choice of priors and Markov chain Monte Carlo settings
☒	☐	For hierarchical and complex designs, identification of the appropriate level for tests and full reporting of outcomes
☒	☐	Estimates of effect sizes (e.g. Cohen's d, Pearson's r), indicating how they were calculated

Our web collection on statistics for biologists contains articles on many of the points above.

Software and code

Policy information about availability of computer code

Data collection	cryo-EM data were collected by Thermo Fisher Scientific EPU. Cross-sectional TEM images were collect by DigitalMicrograph. Fluorescence images were collected by Olympus FV31S. The sperm motility was assessed with Hamilton Thorne's Ceros II system. Tracheal cilia beat frequency movies was recorded by Olympus cellSens Imaging Software. Label-free quantification and protein identification were conducted using Proteome Discoverer 2.5 for database searching. Label-free quantification was conducted using MSFragger via FragPipe v22.0, MSFragger 4.1, and Philosopher 5.1.1.
Data analysis	cryo-EM data were analyzed by RELION-5, PHENIX v1.20.1 and COOT v0.9.8. Other Image and movies were analyzed using Fiji v2.9.0.The GO and KEGG enrichment analyses of differentially expressed proteins were performed using an online server (https://metascape.org). Heatmaps were generated using the Seaborn package in Python.

For manuscripts utilizing custom algorithms or software that are central to the research but not yet described in published literature, software must be made available to editors and reviewers. We strongly encourage code deposition in a community repository (e.g. GitHub). See the Nature Portfolio guidelines for submitting code & software for further information.

Data

Policy information about availability of data

All manuscripts must include a data availability statement. This statement should provide the following information, where applicable:

- Accession codes, unique identifiers, or web links for publicly available datasets
- A description of any restrictions on data availability
- For clinical datasets or third party data, please ensure that the statement adheres to our policy

All source data were provided. The cryo-EM density maps have been deposited into the Electron Microscopy Data Bank under accession numbers EMD-64679, EMD-64623, EMD-64624,EMD64625, and EMD-64626. The coordinate has been deposited into the Protein Data Bank with accession number 9V10. Mass spectrum data have been deposited to ProteomeXchange under accession numbers PXD-073076

Research involving human participants, their data, or biological material

Policy information about studies with human participants or human data. See also policy information about sex, gender (identity/presentation), and sexual orientation and race, ethnicity and racism.

Reporting on sex and gender	no
Reporting on race, ethnicity, or other socially relevant groupings	no
Population characteristics	no
Recruitment	no
Ethics oversight	no

Note that full information on the approval of the study protocol must also be provided in the manuscript.

Field-specific reporting

Please select the one below that is the best fit for your research. If you are not sure, read the appropriate sections before making your selection.

[x] Life sciences [ ] Behavioural & social sciences [ ] Ecological, evolutionary & environmental sciences

For a reference copy of the document with all sections, see nature.com/documents/nr-reporting-summary-flat.pdf

Life sciences study design

All studies must disclose on these points even when the disclosure is negative.

Sample size	All sample sizes are included in the figure legends and methods
Data exclusions	In cryo-EM processing, the bad particles were discarded during multiple round classification. This is widely used and accepted in the cryo-EM field. For all other datasets, no data points were excluded from the analysis.
Replication	All biochemical experiments were performed independently at least three times with similar results, as described in the method and figure legends.
Randomization	For calculation of the Fourier Shell Correlation(FSC), the particles were randomly split into two halves using RELION software. Spermatozoa were from multiple randomly-chosen fields were used in each experiment.
Blinding	Blinding was not applicable to the mouse experiments, as mice were grouped based on genotype. However, all analyses adhered to pre-established protocols to minimize bias.

Reporting for specific materials, systems and methods

We require information from authors about some types of materials, experimental systems and methods used in many studies. Here, indicate whether each material, system or method listed is relevant to your study. If you are not sure if a list item applies to your research, read the appropriate section before selecting a response.

Materials & experimental systems

n/a	Involved in the study	
[ ]	[x]	Antibodies
[x]	[ ]	Eukaryotic cell lines
[x]	[ ]	Palaeontology and archaeology
[ ]	[x]	Animals and other organisms
[x]	[ ]	Clinical data
[x]	[ ]	Dual use research of concern
[x]	[ ]	Plants

Methods

n/a	Involved in the study	
[x]	[ ]	ChIP-seq
[x]	[ ]	Flow cytometry
[x]	[ ]	MRI-based neuroimaging

Antibodies

Antibodies used

rabbit anti-TEKT1 (BS76194, Bioworld), rabbit anti-TEKT5 (BS74841,Bioworld), mouse anti-β-actin (A3854, Sigma), mouse anti-acetylated tubulin (T7451, Sigma) , rabbit anti-RSPH9 (PA5-113359, Invitrogen), rabbit anti-DNAH1 (PA5-57826, Invitrogen), rabbit anti-DNAH17 (24488-1-AP, Proteintech), rabbit anti-CFAP21 (PA5-113157, Invitrogen), rabbit anti-SPAG6 (HPA038440, Sigma), Goat anti-rabbit or anti-mouse secondary antibodies conjugated to Alexa Fluor 488 or Alexa Fluor 647 (ab150077,ab150113,ab150079,ab150115, Abcam). Rabbit polyclonal antibody generated against DUSP21 and Rabbit monoclonal antibody generated against TSSK6 were produced by HUABIO (Hangzhou, Zhejiang, China). The synthesized peptide of DUSP21 (CIFPSQATQQDNIY) conjugated to KLH and the purified full-length protein of TSSK6 were used to immunize rabbits.

Validation

All commercially available primary antibodies have been validated by the manufactures.

Animals and other research organisms

Policy information about studies involving animals; ARRIVE guidelines recommended for reporting animal research, and Sex and Gender in Research

Laboratory animals

All mice used in this study were on a C57BL/6J background. Tekt1-/- mice (Strain NO. T030593), Tekt5-/- mice (Strain NO. T035937), Dusp21-/y mice (Strain NO. T035080) and Tssk6-/- mice (Strain NO. T034984) were purchased from GemPharmatech (Nanjing, China). The mice were maintained in barrier facilities with strictly controlled macroenvironment, including a temperature of 20-26℃, humidity ranges of 40-70%, and a 12-hour light/12-hour dark cycle.

Wild animals

no

Reporting on sex

Both female and male mice were used in this study

Field-collected samples

no

Ethics oversight

All animal studies and experiments were conducted in compliance with institutional guidelines and approved by the Institutional Animal Care and Use Committee (IACUC) at Westlake University (Hangzhou, China).

Note that full information on the approval of the study protocol must also be provided in the manuscript.

Plants

Seed stocks

no

Novel plant genotypes

no

Authentication

no
